# Traumatic Anterior Dislocation of Ocular Cataract Lens

**DOI:** 10.5811/cpcem.2021.7.52725

**Published:** 2021-08-31

**Authors:** Tanvi Shirke, Kyle Wilcox, Thuyvi Luong

**Affiliations:** Wellspan York Hospital, Department of Emergency Medicine, York, Pennsylvania

**Keywords:** traumatic lens dislocation, anterior lens dislocation, lens dislocation, retinal detachment, point-of-care ultrasound, POCUS

## Abstract

**Case presentation:**

A 33-year-old male presented to the emergency department following a motor vehicle collision with complaints of right eye pain after hitting his head on the steering wheel. Point-of-care ultrasound (POCUS) revealed retinal detachment and an anterior lens dislocation.

**Discussion:**

Lens dislocations following blunt head trauma can often be diagnosed using POCUS. Anterior ocular lens dislocation is a rare but vision-threatening result of head trauma. This case highlights how POCUS can facilitate early detection of ocular pathology, such as lens dislocation, and improves patient outcomes.

## CASE PRESENTATION

A 33-year-old man with a history of blindness in his right eye from a congenital cataract presented to the emergency department with blunt head trauma sustained during a motor vehicle collision. He complained of right eye pain and foreign body sensation. On examination, a round white object was visualized within the anterior chamber; his head was otherwise atraumatic. The patient stated that the white spot had been present prior to the accident but had now changed in size and appearance, noting that the spot had enlarged following his injury.

**Image 1:**
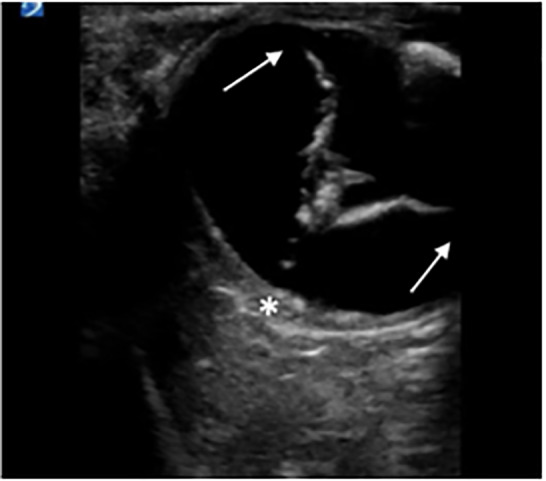
Right eye transverse view showing retinal separation with attachment at the level of the optic disc (*) and ora serrata (arrows).

**Image 2:**
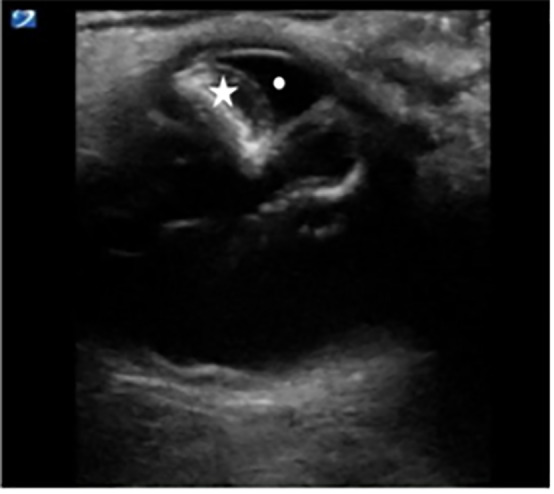
Right eye sagittal view depicting echogenic lens indicative of cataract (star), as well as subluxation of lens into anterior chamber (white dot) indicative of anterior dislocation of lens.

**Image 3:**
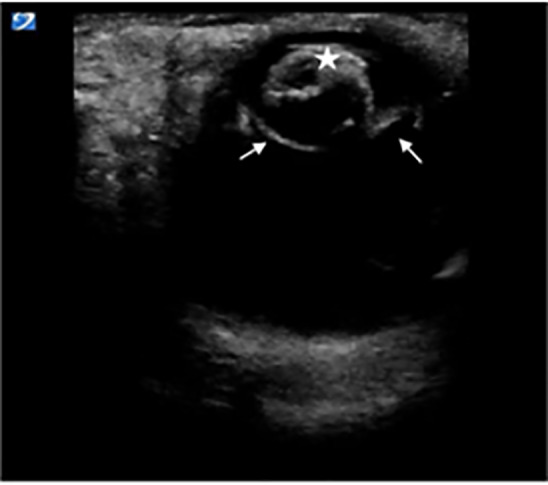
Right eye transverse view showing lens (star) displaced anterior to the iris and ciliary bodies (arrows).

Fluorescein staining showed no abnormalities, and intraocular pressures were normal (18 millimeters mercury [mm Hg] right eye; 20 mm Hg left eye). Light perception was not present. Slit lamp examination demonstrated a round, white-speckled object in the dependent portion of the anterior chamber. Bedside ocular ultrasonography revealed a retinal detachment and an anterior dislocation of a cataract lens through the iris (Images 1–3). Ophthalmology was consulted; anterior lens dislocations are considered an ocular emergency as they can result in acute angle-closure glaucoma and corneal edema; however, given the patient’s previous right-sided blindness they recommended next-day follow-up for operative repair.

## DISCUSSION

Crystalline lens dislocation, or ectopia lentis, occurs primarily after blunt head trauma.^2^ Lens dislocations occur as a result of damage to the zonular fibers of the ciliary body, which hold the lens in place. Disruption of the zonular fibers may result in either a partial or complete lens dislocation.^3^ In a partial dislocation, the lens partially maintains its position behind the iris. In a complete luxation, the lens is found completely outside of the hyaloid fossa.^3^ Often, the lens is found within the vitreous of the posterior compartment of the eye. Very rarely is it found within the anterior chamber.^4^,^5^

Patients can present with eye pain and visual changes ranging from light distortion to loss of vision.^1^,^4^ If unrecognized or untreated, anterior dislocations can block the anterior chamber and trabecular meshwork causing elevated intraocular pressures, resulting in glaucoma, pupillary block, and corneal edema.^1^,^4^ Therefore, they are considered vision-threatening emergencies.

**CPC-EM Capsule**

What do we already know about this clinical entity?

*Anterior ocular lens dislocation is a potential result of blunt head trauma. If unrecognized, it can block the anterior chamber causing elevated intraocular pressures.*

What is the major impact of the images?

*The sonographic appearance of anterior lens dislocation has not been well described in emergency medicine literature. These are some of the first reported images.*

How might this improve emergency medicine practice?

*Because anterior lens dislocation can result in vision loss, accurate diagnosis is important. Point-of-care ultrasound can be used to diagnose lens dislocations.*

Ultrasonography can aid in the diagnosis of all types of lens dislocations and assess for additional ophthalmologic pathology, including retinal detachment and vitreous hemorrhage.^4^ Point-of-care ultrasonography (POCUS) can be used to make a rapid diagnosis without the delays associated with magnetic resonance imaging or the ionizing radiation of computed tomography. Diagnosis of posterior lens dislocation by POCUS has been previously described in the literature; however, the sonographic appearance of the rarer anterior lens dislocation has not been well documented.^5^ This case provides some of the first reported ultrasound images of an anterior lens dislocation.
